# AI-Guided Discovery of Oncogenic Signaling Crosstalk in Tumor Progression and Drug Resistance

**DOI:** 10.32604/or.2026.076157

**Published:** 2026-04-22

**Authors:** Edward Sutanto, Rinni Sutanto, Sara Velichkovikj, Nikola Hadzi-Petrushev, Mitko Mladenov, Dimiter Avtanski, Radoslav Stojchevski

**Affiliations:** 1 CUNY School of Medicine, The City College of New York, New York, NY, USA; 2 New York Institute of Technology College of Osteopathic Medicine, Glen Head, NY, USA; 3 Office of Clinical Research, Lenox Hill Hospital, Northwell Health, New York, NY, USA; 4 Faculty of Natural Sciences and Mathematics, Institute of Biology, Ss. Cyril and Methodius University, Skopje, North Macedonia; 5 Friedman Diabetes Institute, Lenox Hill Hospital, Northwell Health, New York, NY, USA; 6 Donald and Barbara Zucker School of Medicine at Hofstra/Northwell, Hempstead, NY, USA; 7 Institute of Bioelectronic Medicine, Feinstein Institutes for Medical Research, Manhasset, NY, USA; 8 Institute of Molecular Medicine, Feinstein Institutes for Medical Research, Manhasset, NY, USA

**Keywords:** Artificial intelligence, machine learning, oncogenic signaling pathways, neural networks, tumor progression, drug resistance

## Abstract

The rapid growth and accessibility of artificial intelligence (AI) and machine learning (ML) have opened many avenues to revolutionize biomedical research, particularly in oncogenesis. Oncogenesis is a hallmark process in the development of cancer, involving the amplification of proto-oncogenes and the subsequent dysregulation of molecular signaling networks. These pathways—including the RAS/RAF/MEK/ERK, PI3K-AKT, JAK-STAT, TGF-β/Smad, Wnt/β-Catenin, and Notch cascades—have been studied extensively in isolation, with major strides achieved in understanding how they drive cancer. However, there are still many considerations regarding how these networks interact. Ongoing studies show that crosstalk among these pathways occurs through feedback loops, shared intermediates, and compensatory activation, creating a complex network that enables tumor cells to adapt and metastasize. New developments in AI and ML have enabled modeling and prediction of these interactions for pathway discovery, mapping oncogenic crosstalk, predicting drug resistance and therapeutic responses, and complex data analysis. Novel technologies such as feature selection algorithms and convolutional neural networks have demonstrated immense translational potential to bridge computational predictions in cancer genomics with clinical applications. Similar models have also proven useful for learning from genomic datasets and reducing multidimensionality in heterogeneous multiomics data. As current AI/ML approaches continue to develop, it is also important to consider the limitations of batch effects, model generalizability, and potential bias in training datasets. This review aims to integrate the most recent AI and ML applications in uncovering the hidden interactions within oncogenic networks that drive tumorigenesis, heterogeneity, and resistance to therapies. Moreover, this review aims to synthesize the functionality of emerging computational methods that elucidate these insights, as well as the transformative implications of AI-guided systems biology on precision oncology and combinatorial therapies.

## 1 Introduction

Cancer arises from the progressive breakdown of normal cellular signaling and aberrant cell growth, reflected in the multi-step molecular process of oncogenesis [1]. Proto-oncogenes are the first regulatory factors in the process responsible for normal cell growth and proliferation, as they play a role in the production of growth factors, growth factor receptors, signal transduction molecules, and nuclear transcription factors [2]. In oncogenesis, these proto-oncogenes undergo genetic variations/mutations, gene duplication, epigenetic changes, or chromosomal rearrangements, potentially converting them to oncogenes [3]. The activation of proto-oncogenes into oncogenes leads to the hyperactivation and dysregulation of many signaling cascades, including the RAS/RAF/MEK/ERK, PI3K/AKT, JAK-STAT, TGF-β/Smad, Wnt/β-Catenin, and Notch pathways, which can promote uncontrolled proliferation and potentially cause cancer [3,4].

Decades of extensive research have shown that, when dysregulated, these key signaling pathways can become self-sustaining circuits that contribute to tumorigenesis and metastasis. However, cancers rarely have a dysfunctional linear signaling pathway, but rather an adaptive, interdependent network [5]. Pathway crosstalk and dynamic feedback loops have been shown to have major implications for drug sensitivity and dose-response, as well as for the initiation and progression of epithelial-to-mesenchymal transition (EMT), a key step in metastasis in which sessile epithelial cells change into motile mesenchymal cells [6,7]. Uncovering the mechanisms behind pathway redundancy remains a barrier and the key to understanding how tumors develop and adapt to therapies in oncology.

Artificial intelligence (AI) refers to algorithms or machines that mimic human intelligence; machine learning (ML), a subfield of AI, involves computers using statistical techniques to discover hidden patterns in data [8]. The use of AI and ML in biomedicine has seen exponential growth with a wide range of applications spanning diagnosis, prognosis, treatment, surgery, and drug discovery [9]. Recent advances in AI and ML are transforming oncology by enabling the integration of vast datasets, including multiomics and imaging data, to model oncogenic signaling [10]. Moreover, these AI-driven frameworks can identify hidden relationships among pathways, reveal predictive biomarkers of therapeutic response, and simulate mechanisms that are difficult to capture experimentally [8]. The convergence of AI and oncogenic signaling research paves the way for a new era of precision oncology that can overcome the complexities of tumor signaling.

This review uses a narrative methodology to summarize the latest breakthroughs in the use of AI and ML models for oncogenic signaling crosstalk and their implications for tumor development, metastasis, and drug resistance. A comprehensive search across major databases, including PubMed, Embase, Scopus, and Web of Science, was conducted. The search strategy included keywords such as “machine learning”, “deep learning”, “neural networks”, “artificial intelligence”, “oncogenic signaling pathway”, “crosstalk”, “pathway co-activation”, “oncogenesis”, “network interaction”, and “drug resistance”, among others. Other articles were also independently identified through reference lists and the latest review articles. Following screening, articles were included based on their relevance to AI and ML in cancer signaling interactions, then thematically sorted and assessed for their value and limitations. Accordingly, this review aims to synthesize the most recent advances in AI/ML approaches in modeling oncogenic signaling crosstalk, as well as their translational implications on tumor heterogeneity, drug resistance, and the development of precision and combinatorial cancer therapies.

## 2 Foundations of Oncogenic Signaling Crosstalk

### 2.1 Overview of Major Signaling Pathways

Mutations in different oncogenic pathways and signaling cascades play a major role in metabolic reprogramming, an essential factor in cancer development and progression [11]. Understanding how they function and their downstream effects is crucial to identifying potential therapeutic interventions [4].

The RAS/RAF/MEK/ERK, or the mitogen-activated protein kinase (MAPK) pathway, is the most frequently affected signaling pathway in oncogenesis [12]. The pathway begins with receptor tyrosine kinases (RTKs), where ligand binding induces dimerization and autophosphorylation of their tyrosine residues, providing a binding site for a protein complex that recruits and activates Ras, initiating a cascade of phosphorylation of dual-specificity kinases [13]. Produced ERK/MAPK enters the nucleus and subsequently activates transcription factors such as Elk-1 and c-Myc downstream [14]. The pathway plays a vital role in anti-apoptotic regulation and degradation of extracellular matrix proteins, and its dysregulation leads to cancer metastasis and angiogenesis [12].

The PI3K-AKT-mTOR signaling pathway is another pathway that is often dysregulated in many cancers. Like the MAPK pathway, the PI3K-AKT-mTOR pathway involves the activation of RTKs. The conversion of intramembrane phosphatidylinositol 4,5-bisphosphate (PIP_2_) into phosphatidylinositol 3,4,5-triphosphate (PIP_3_) provides a docking site for AKT and PDK1, where cross-phosphorylation activates AKT and releases it into the cytosol [15]. AKT regulates cell survival and growth by inhibiting pro-apoptotic factors such as Bad, activating mTORC1, and inhibiting glycogen synthase kinase 3 (GSK-3) [16,17]. Oncogenesis is heavily influenced by aberrant expression of many components of this pathway, including the induction of oncogenes upstream of PI3K, kinase amplifications, and mutations in the AKT oncogene [17,18].

The JAK/STAT pathway functions similarly to the RTK-associated pathways; however, it relies on cytokine receptors that lack intrinsic autophosphorylation and instead use associated proteins [19]. Cytokine binding induces dimerization, in which JAK proteins cross-phosphorylate each other, which then phosphorylate STAT, releasing them into the cytosol, where they dimerize and translocate to the nucleus to activate transcription factors [19]. The JAK/STAT pathway has gained recognition for its role in upregulating EMT-inducing transcription factors, including Snail, ZEB1, JUNB, and Twist1, during oncogenesis [20].

The TGF-β/Smad cascade operates using a unique serine/threonine kinase mechanism involving heterodimeric complexes [21]. The binding of TGF-β to type II receptors prompts the recruitment and dimerization with the type I receptor [21]. Here, the type II receptor phosphorylates the type I receptor, which, in its activated state, recruits the transcription factors R-Smads (Smad2/Smad3) and the Co-Smad (Smad4). This transcription complex translocates to the nucleus to regulate gene expression [21,22]. In cancer, multiple cell types produce and respond to TGF-β, conferring it immense tumor-promoting effects [23].

The Wnt/β-Catenin pathway regulates cell proliferation, differentiation, and stem cell maintenance. β-Catenin acts as both a structural protein at cell-cell junctions and as a transcriptional coactivator [24]. In the absence of the Wnt ligand, β-Catenin is continuously degraded by the destruction complex consisting of APC, Axin, GSK3β, and CK1 through ubiquitination and proteasomal degradation [25]. The presence and binding of Wnt ligands to the Frizzled (FZD) receptor inhibits the destruction complex, allowing β-Catenin to accumulate and translocate to the nucleus, where it partners with transcription factors to activate oncogenes such as *MYC* and *CCND1* [25,26].

The Notch pathway is highly conserved and plays important roles in development, homeostasis, cellular proliferation, differentiation, and apoptosis [27]. It involves intercellular signaling interactions where ligands, such as Delta-like or Jagged, bind to the Notch receptor on an adjacent cell [28]. A series of proteolytic cleavages releases the Notch intracellular domain (NICD) into the nucleus, where it activates the HES and HEY transcription factors [28]. Extensive research has revealed the prominent role of the dysregulated Notch signaling pathway in EMT, angiogenesis, metabolic reprogramming, and regulation of the tumor microenvironment [29].

The NF-κB/TNF pathway is responsible for the transcriptional regulation of genes involved in immune and inflammatory pathways, cell survival, and proliferation [30]. The TNF receptor superfamily activates the noncanonical NF-κB, which also regulates the activation, differentiation, and effector function of regulatory T cells as well as the induction of apoptosis inhibitors such as Bcl-2, Bcl-XL, c-IAP1, cIAP2, and c-FLIP [31]. As such, aberrant NF-κB/TNF has been well established to contribute to tumor cell proliferation, survival, metabolism, metastasis, tumor angiogenesis, and therapy resistance [31].

The Hedgehog pathway is a complex signaling cascade that is crucial in embryonic development and tissue homeostasis, specifically regulating the proliferation and differentiation of adult stem cells [32]. The pathway includes three ligands—Sonic Hedgehog, Indian Hedgehog, and Desert Hedgehog—which are responsible for the central nervous system development, endochondral ossification, and gonadal development, respectively [33]. Extensive research has shown that both autocrine and paracrine aberrant activity of the Hedgehog signaling pathway is responsible for the initiation, growth, and drug resistance of various neoplasms, including medulloblastoma, basal cell carcinoma, and other solid hematological tumors [33,34].

The Hippo signaling pathway is a conserved signaling network that regulates numerous physiological processes, including cell proliferation, differentiation, and survival, as well as organ development and homeostasis [35]. As a kinase cascade, the Hippo pathway comprises MST1/2, SAV1, LATS1/2, YAP, and TAZ [36]. Unlike most pathways regulated by transmembrane receptors and their cognate ligands, the Hippo pathway is controlled by the tissue microenvironment [35,37]. When the pathway is inactive, its effectors, YAP and TAZ, translocate to the nucleus, activating genes for cell proliferation, survival, and stemness, thereby contributing to liver, colorectal, and, most notably, lung cancers, including mesothelioma [37].

### 2.2 Mechanisms of Pathway Interaction and Feedback

**Figure 1:**
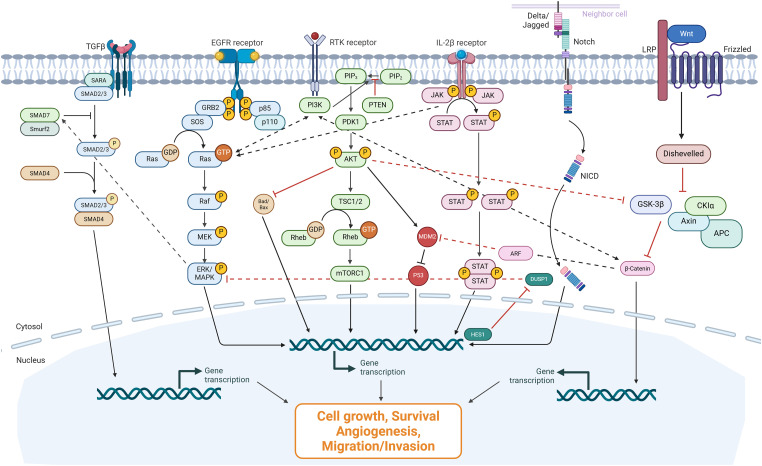
Key oncogenic signaling pathways and crosstalk mechanisms. Left: The TGF-β/SMAD is responsible for the transcriptional regulation of growth and differentiation genes, with SMAD being translocated to the nucleus downstream. ERK serves as a regulator of this pathway by activating SMAD7, which inhibits the phosphorylation of SMAD2/3. Middle: The RAS/RAF/ERK/MAPK, PI3K/AKT, and JAK/STAT pathways converge to regulate cell growth, survival, angiogenesis, and migration/invasion in oncogenic settings. PI3K, responsible for the conversion of PIP_2_ to PIP_3_, exhibits direct crosstalk with GTP-bound Ras as well as β-Catenin. AKT serves as an activator of MDM2, which inhibits p53 downstream. Right: The Notch signaling exhibits crosstalk with the MAPK/ERK pathway through HES1, which serves as an inhibitor of DUSP1, an inhibitor of ERK. Within the destruction complex, GSK-3β inhibits β-Catenin, which activates ARF and inhibits MDM2, which connects to the PI3K/AKT pathway. Interrupted lines represent well-established crosstalk mechanisms. Created in BioRender.com. Stojchevski R. (2025) https://BioRender.com/ei3zp1f [40].

As oncogene research continues to see immense progress, several mechanisms of signaling crosstalk have been established, including feedback loops, cross-inhibition, cross-activation, and pathway convergence [38]. These mechanisms allow parallel pathways to dynamically regulate one another, which, in a normal context, preserves cellular homeostasis but can induce tumorigenesis in an aberrant setting [39]. This feedback, convergence, and compensatory activation occur across many of the most crucial signaling cascades, as shown in Fig. 1.

The MAPK/ERK and PI3K/AKT pathways are two of the most important signaling cascades and are often the most dysregulated in oncogenesis [41]. Studies have shown that the MAPK/ERK and PI3K/AKT pathways are highly interdependent, with aberration of one pathway amplifying the other [42]. A study conducted on basal-like breast cancer found that either loss of *PTEN* or *KRAS* activation alone does not produce detectable tumors; however, co-activation of both pathways results in aggressive tumorigenesis [42]. Moreover, simultaneous inhibition of these two signaling pathways holds strong therapeutic potential. For instance, Dreaden et al. (2015) [43] demonstrated that systemic administration of tumor-targeted layer-by-layer nanoparticles loaded with a synergistic combination of MEK and PI3K small-molecule inhibitors enhanced apoptosis, reduced adaptive resistance, and stabilized breast tumors. In contrast, untargeted nanoparticles loaded with the dual inhibitors or free-drug combinations resulted in tumor progression. Activation of the PI3K/AKT pathway following MAPK inhibition has also been observed in BRAFV600E melanoma, as well as in MAPK-driven resistance to PI3K inhibitors in adult acute lymphoblastic leukemia [44,45].

The Wnt/β-catenin and PI3K/AKT pathways play important roles in the development of malignant phenotypes in cancer, with bidirectional regulation that drives growth, survival, and therapeutic resistance [46]. One of the primary ways in which these two pathways interact is through AKT-mediated phosphorylation of GSK3β, which stabilizes β-catenin and amplifies Wnt transcriptional activity, enabling nuclear β-catenin to reciprocally promote PI3K signaling by upregulating transcription of growth-factor receptors [47,48]. Recently, studies have demonstrated that crosstalk between PI3K/AKT and multiple signaling pathways, including the Wnt/β-Catenin cascade, can promote glioblastoma progression and reduce glioma cell sensitivity [49]. Collectively, this shows that Wnt/β-catenin and PI3K/AKT bidirectional integration provides a rationale for combination targeting.

The Notch and PI3K/AKT signaling cascades exhibit strong oncogenic crosstalk in which Notch activation by ligands such as JAG1 stimulates PI3K/AKT signaling. At the same time, AKT stabilizes the Notch intracellular domain (NICD) [29]. Clinically, Notch signaling has been shown to interact with PI3K/AKT to maintain survival, DNA repair, and angiogenesis in glioblastoma, thereby increasing the likelihood of therapy resistance and tumor recurrence [50]. Targeting these two pathways has also been therapeutically relevant, as a study conducted in acute lymphoblastic leukemia showed that dual inhibition of Notch1 and PI3K/AKT resulted in synergistic cytotoxicity and apoptosis in resistant cell lines [29,51].

### 2.3 Clinical Implications of Signaling Convergence

The interaction among these major signaling pathways is further highlighted by the promising results of combination therapies that simultaneously target multiple pathways. Studies have shown that aberrant activation of the PI3K/AKT and MAPK/ERK pathways results in strong compensatory crosstalk, rendering single-pathway inhibition largely ineffective. In lung tumor-derived cell lines, compensatory feedback loops have been identified in which inhibition of ERK induces AKT activation, and *vice versa* [52]. In one study investigating metastatic melanoma, inhibiting only one cascade—either the MEK inhibitor trametinib or the PI3K inhibitor buparlisib—led to reciprocal pathway activation and adaptive resistance in the tumor [53]. However, their combined therapy was shown to suppress proliferation while increasing apoptosis by upregulating pro-apoptotic proteins, such as Bad and cleaved caspase-3, and downregulating anti-apoptotic factors, such as Bcl-2 [53]. Furthermore, a similar result was found in a study investigating Merkel cell carcinoma. A study by Temblador et al. (2022) [54] showed that dual inhibition of mTORC1/2 and MEK1/2 with MLN0128 and trametinib, respectively, synergistically produced stronger antiproliferative effects compared to monotherapies.

Another study showed that the mTOR inhibitor temsirolimus was effective in treating relapsed/refractory multiple myeloma when combined with additional pathway-targeting therapies [55]. Multiple myeloma cell lines showed increased activity in the PI3K and mTOR pathways, and mTOR inhibition with temsirolimus reduced phosphorylation of key downstream PI3K pathway molecules [56]. Combining temsirolimus with trametinib, a MEK inhibitor acting on the MAPK/ERK pathway, amplified activity in decreasing the proliferation of multiple myeloma, when compared to temsirolimus and trametinib monotherapies alone [56]. The synergistic activity demonstrated by this combination therapy further supports the existence of pathway crosstalk in multiple myeloma and offers a promising potential for effective treatment. These studies underscore a key principle that signaling convergence, in this case the PI3K/AKT and MAPK pathways, represents both a mechanism of resistance and a therapeutic vulnerability.

Other clinical studies have demonstrated the need to treat overlapping signaling pathways in tumors that exhibit adaptive resistance. A large Phase I clinical trial showed that patients with PI3K/AKT and MAPK/ERK coactivation, particularly those with KRAS or BRAF alterations, experienced tumor regression with dual pathway inhibition compared with a single agent [57]. Although combined therapy was associated with increased toxicity, the findings underscore the biological and clinical necessity of identifying pathway interactions. Another study evaluating the combined inhibition of PI3K/AKT and MAP/ERK using buparlisib and trametinib, respectively, saw meaningful antitumor activity, specifically in KRAS-mutant ovarian cancer with multiple solid tumors [58]. Similarly, a study by Bardia et al. (2020) showed that dual PI3K/AKT and MAPK/ERK inhibition produced partial responses in tumors, as evidenced by downregulation of pERK and pS6 [59]. These studies emphasize that therapeutic efficacy depends on dynamic, compensatory signaling networks, creating analytical complexity that is well-suited to AI/ML approaches for uncovering interaction patterns in large datasets. Most importantly, the nonlinear, compensatory, and highly context-dependent nature of these pathway interactions often exceeds the current capacity of traditional reductionist models, calling for AI/ML frameworks that are more powerful and capable of integrating large-scale data processing to capture micro-signaling behaviors and guide combinational therapies.

## 3 AI/ML Technologies Applied to Pathway Discovery

AI is a broad umbrella term that encompasses the field of computer science in which computational systems are designed to mimic human intelligence and perform tasks such as pattern recognition and decision-making [8]. There are many subsets of AI, most notably ML, where computers carry out predetermined tasks and combine statistical techniques for pattern recognition in data [8]. ML techniques then extend to deep learning, supervised learning, and unsupervised learning [60]. Deep learning employs neural networks to address problems that are difficult to solve with traditional machine learning techniques, enabling intelligent analysis of large-scale data [60]. Meanwhile, supervised learning uses labeled data to train models to predict specific outcomes, while unsupervised learning uses unlabeled data to discover hidden patterns and extract generative features [61].

### 3.1 Supervised and Unsupervised Learning in Genomic Datasets

To accurately analyze and predict future data, AI models must use ML to learn patterns from past datasets. ML falls into two major categories: supervised and unsupervised, each with its own strengths when applied to cancer genomics. Supervised ML relies on identifying patterns from known classes (labeled genomic data points), whereas unsupervised learning discovers commonalities from unknown classes (unlabeled data points) [62]. Unsupervised learning is achieved primarily through clustering algorithms that identify subgroups within a data population, which have been shown to successfully uncover disease-related gene clusters [63,64]. Supervised learning algorithms, including classification and regression algorithms, generally aim to predict the correct class, i.e., the label or category [63]. In genomics, supervised learning classification algorithms have been used in diagnostics to predict the presence of Alzheimer’s disease and schizophrenia, as well as to identify predictive and prognostic biomarkers in cancer [65–67].

Deep learning, a subset of supervised ML, is a multi-layer neural network algorithm that extracts patterns from multidimensional data. The neural network algorithm, therefore, enables more complex analysis of diverse data types, such as genomic, histopathologic, and transcriptomic data that inform cancer diagnosis or identification [68]. Recently, deep learning algorithms have been developed to analyze and predict patient survival rates by integrating data on signaling pathways and their effects on cancer progression and drug resistance [69]. The study developed a biologically meaningful deep neural network, DeepSigSurvNet, to analyze 1967 genes from 46 major signaling pathways in four different cancer types [69]. Results showed that certain signaling pathways had stronger relevance scores among the cancers; for example, p53 and mTOR signaling pathways were shown to be most implicated in poorer survivability of glioblastoma, breast cancer, and skin cutaneous melanoma, while MAPK, ErbB, Ras, Rap1, and JAK/STAT signaling pathways had lower relevance scores, despite being known to play significant roles in cancer and tumorigenesis [69]. The performance of the proposed model was evaluated using the c-index metric, which was compared with a random forest model and evaluated across multiple sampling ratios to assess robustness to heterogeneity in The Cancer Genome Atlas (TCGA) dataset [69]. Lower c-index values in the test datasets than in the proposed model indicated that the deep learning model was statistically powerful and robust [69]. The significance of these pathways in survival prediction is biologically plausible, as they govern stress responses, metabolic control, and cell-cycle checkpoints that influence tumor adaptability. Moreover, the lower activity of MAPK/ERK and JAK/STAT indicates that, in certain contexts, tumors may rewire toward metabolic and DNA-damage networks [70]. Importantly, these pathway relevance scores reflect predictive associations with patient outcomes rather than direct evidence of causal signaling interactions. Using deep learning to uncover signaling pathways associated with poor survival may have significant clinical potential for tailoring treatment to improve patient outcomes.

### 3.2 Feature Selection and Dimensionality Reduction in Multiomics

Multiomics in cancer research has helped provide an integrated, comprehensive understanding of cancer. However, the heterogeneous nature of the data, including genomics, transcriptomics, and proteomics, may pose challenges for applying AI and ML technologies. With the increase in multidimensionality of cancer data available for analysis, methods have been developed to reduce dimensionality and data noise for more accurate prognosis and prediction. Most importantly, the primary objective is to identify the most biologically informative features, such as genes, proteins, or phosphosites, from noisy, high-dimensional multi-omics datasets to improve model performance, interpretability, and translational relevance.

Feature selection is the process of identifying relevant features and discarding irrelevant ones, which supports the learning process in ML algorithm development [71]. Pattern recognition for algorithm development generally involves three phases: data acquisition, dimensionality reduction, and classification, with the first phase yielding a wide range of features that need to be filtered and refined for relevance [72,73]. Feature selection and dimensionality reduction can be achieved through both supervised and unsupervised methods [74]. A study examined five commonly used supervised feature selection algorithms across datasets for Acute Myeloid Leukemia (AML) and compared their classification performance, redundancy rates, and representation entropies [75]. One such dataset was the Paradigm IPLs, which represented a pathway activity dataset for AML tumor samples. The application of these feature selection algorithms identified three gene signatures associated with pathway activity: *BMP4*, *STXBP1*, and *LEP*, which have been shown to play significant roles in the MAPK, JAK/STAT, and PI3K pathways [75–77]. Validation using 10-fold cross-validation across classifiers such as C4.5, Naïve Bayes, KNN, and AdaBoost showed that VWMRmR-based models achieved the highest cross-validated accuracy, with robustness confirmed by classification accuracy, redundancy rate, and representation entropy rather than dataset-specific overfitting [75]. More importantly, these genes occupy biologically meaningful positions: *BMP4* interfaces with MAPK/ERK and TGF-β signaling, while LEP influences PI3K/AKT-mediated metabolic regulation. These findings suggest that feature selection can effectively highlight nodes involved in cross-pathway integration rather than isolated signaling.

Feature selection algorithms generally fit into three categories: filter methods, wrapper methods, and embedded methods [78–80]. Filter methods assess data for intrinsic properties to evaluate relationships between features and the target, whereas wrapper methods evaluate the capabilities of a performance model using different subsets [81]. Embedded methods integrate the selected features into the model training process to identify the significant features [81]. Many studies have shown the efficacy of various feature selection algorithms in identifying biological markers across diverse cancer subtypes. For example, feature selection algorithms have been particularly useful for breast cancer, which exhibits distinct molecular subtypes such as Luminal A, Luminal B, HER2-enriched, and basal-like (similar to triple-negative breast cancer, TNBC) [81].

### 3.3 Use of Neural Networks, Graph-Based Models, and Ensemble Methods

Various AI and ML models have been developed to best fit different data types. For example, neural networks have been modeled after the human brain, with layers of interconnected “nodes” representing neurons. Neural networks typically consist of three layers: an input layer, multiple hidden layers, and an output layer [82]. The input layer receives raw data, in the form of both images and text, while the deep layers perform data analysis using computational algorithms for pattern recognition and extraction. Finally, the output layer delivers the final prediction or prognosis [83]. These networks offer a wide range of potential across many facets of cancer genomics and signaling (Table 1).

**Table 1:** Prevalent applications of neural-network architectures in oncogene signaling research.

Neural Network Type	Mechanism	Primary Role/Typical Application	Dataset Analysis Models	Key Advantages, Limitations	References
Convolutional Neural Network (CNN)	Uses convolution & pooling layers to extract spatial features (i.e., edges, textures) from matrices or images	Maps histologic and transcriptomic features to pathway activations (i.e., EMT, Wnt/β-Catenin)	DeepClassPathway (HPV+ vs. HPV-HNSCC)	Excels in special pattern recognition without manual labeling	[84–86]
Graph Neural Network (GNN)	Learns relationships among data nodes (i.e., genes, proteins) linked by edges representing biological interactions	Models crosstalk and network topology among pathways (PI3K ↔ MAPK, JAK-STAT, Wnt)	CANDELA; SynerGNet; Graph Layer-wise Relevance Propagation	Captures non-Euclidean network structure; reveals cross-pathway signaling	[87–90]
Ensemble Neural-Network Methods	Combines several baseline models to build a bigger single yet more powerful model for consensus prediction	Combines multiple learners for robust multi-pathway gene and signaling discovery	NF-κB/TNF Ensemble	Increases accuracy and robustness across datasets; limited mechanistic interpretability	[91–94]
Feed-Forward/Deep Neural Network (DNN)	Sequential interconnected layers that process input and move to the hidden layer to the output without feedback	Integrates multi-omics data to predict pathway activity or survival	DeepSigSurvNet; PathDeep; DeepGene	Handles nonlinear genomic relationships; interpretable by layer	[69,95–97]
Autoencoder/ Variational Autoencoder (VAE)	An encoder-decoder structure compresses and reconstructs high-dimensional omics data	Reduces dimensionality of multi-omics data before classification or clustering	Moanna	Reduces noise and redundancy; useful for pathway modeling	[98–100]

Note: Abb: Epithelial-to-Mesenchymal Transition (EMT); Human Papillomavirus (HPV); Phosphoinositide 3-kinase (PI3K); Protein Kinase B (AKT); Janus Kinase (JAK); Signal Transducer and Activator of Transcription (STAT); Mitogen-Activated Protein Kinase (MAPK); Head and Neck Squamous Cell Carcinoma (HNSCC); Nuclear Factor kappa-light-chain-enhancer of activated B cells (NF-κB); Tumor Necrosis Factor (TNF).

One of the widely used neural networks is the Convolutional Neural Network (CNN), developed to mimic a complex neural pathway in the visual cortex to identify relevant features without human supervision [101,102]. What sets CNNs apart from other neural networks is their ability to use specialized filters to detect specific patterns in images, such as edges, curves, and textures, which is especially useful for pattern recognition across various histological samples of cancer [103,104]. CNN techniques have also been shown to help isolate signaling pathways in cancers; for example, one study proposed a novel CNN-based classifier, DeepClassPathway, for correlational analysis of human papillomavirus (HPV)-associated head and neck squamous carcinoma. The algorithm was trained using transcriptomic data from over 50 molecular pathways to predict HPV status in patients with head and neck tumors, as HPV-positive and HPV-negative cancers have distinct clinical and molecular properties that affect treatment [84,105,106]. The CNN algorithm consistently identified activation of mutant *KRAS* genes in HPV-positive cancers and *MYC* targets for HPV-negative patients [84]. This algorithm was then verified through cross-validation runs and independent test cohorts, which consistently yielded high, stable performance across repetitions, with Receiver Operating Characteristic-Area Under the Curve (ROC-AUC) and Precision Recall (PR)-AUC values of 0.96 and 0.90, respectively, indicating strong generalizability of the model outside of the sample cohort [84]. Robustness was further assessed through pathway-shuffling experiments and treemap permutations, which showed reproducible Grad-CAM patterns across model ensembles [84].

A specialized form of neural networks, called graph-based methods, or graph neural networks, has been developed to learn from graph-structured data in which data nodes are connected by relationships [107]. While CNN methods can be used for processing images in Euclidean domains, they are unable to handle non-Euclidean domains such as molecular networks in biology; therefore, graph neural networks have been developed to process non-Euclidean domains and close this knowledge gap [108]. A recent study applied graph neural networks to process over 500 molecular network signatures from patients to predict metastasis in breast cancer [89]. Each patient’s molecular signature was represented by a single graph and processed for both metastatic and non-metastatic breast cancer patients. Among all breast cancer patients, graph neural network models identified increased expression of the actin-binding protein cofilin (CFL1) and the *STAT3* gene, which are responsible for tumor cell motility and invasiveness, respectively, and for epithelial-to-mesenchymal transformation [89,109]. The Graph-CNN showed stable performance across repeated 10-fold cross-validation (AUC = 0.83), with Modified National Institute of Standards and Technology (MNIST) dataset sanity checks showing near-perfect accuracy and robustness in validation [89]. This study is particularly valuable for its partial mechanistic support, as it provides experimental grounding—although limited—whereas most studies are fundamentally association-driven analyses. However, it still lacks direct causal testing in signaling mechanisms.

Graph neural network analysis of individual patient profiles also revealed expression of signaling pathway-related genes, such as *SOX4* and *VIM*, in metastatic breast cancer patients, which are associated with cell proliferation and early metastasis formation [89,110–112]. Molecular network analysis of non-metastatic patients revealed upregulation of Ras GTPase-activating protein 1 (RASA1), which has been associated with a favorable prognosis [113]. The study demonstrated that graph-based deep neural networks can be useful for observationally identifying cell signaling pathway genes already associated with breast cancer, and therefore, have the potential to discover new pathways in other cancers as well [113]. These findings were then validated in two independent patient cohorts, orthogonal measurement tools, and long-term follow-up survival analyses, with results remaining significant after multivariate Cox Proportional Hazards regression [113].

Ensemble methods have also been developed as an advanced subset of neural networks that combine multiple algorithms and are useful for classifying different cancer types [114–116]. Recently, new ensemble methods have been developed to investigate NF-κB/TNF signaling pathways in cancer. The study combined 16 algorithms to identify genes associated with the NF-κB/TNF signaling pathways across 16 cancer types [92]. 198 NF-κB/TNF hallmark genes were used in the learning phase, along with an additional 19,671 cancer genes not typically associated with the NF-κB/TNF signaling pathways [92]. The ensemble methods technique successfully identified the known NF-κB/TNF hallmark genes across the given cancer samples, including the highly prevalent *EGR1*, *JUNB*, and *ZNF36* genes, which have been shown to play an important role in tumorigenesis across different cancer types [92,117,118]. More significantly, the ensemble method identified new candidate gene signatures associated with downstream processes of the NF-κB/TNF signaling pathways, specifically the *SRGN*, *CCN2*, *TNFRSF12A*, and *ZFP36L1* genes, which are highly likely to be involved in NF-κB/TNF signaling pathways across various cancers [79]. The ensemble model was validated through large-scale internal hold-out testing, where ROC-AUC comparisons against correlation-based baselines and aggregation across 16 TCGA cancer types showed consistently high AUCs, with >0.75 in most cancers and 0.94 across all 16 cancer types [92]. The biological significance of these AI-predicted features is also supported by their consistent association in established NF-κB/TNF, cytokine, anti-apoptotic, and MAPK/ERK signaling pathways. These signaling pathways are known to be involved in oncogenic interaction, pathway dysregulation, and drug resistance. This consistency in prediction and their association with poor outcomes suggests that AI systems identify biologically meaningful signaling dependencies rather than random correlations, thus highlighting experimentally valid components within these signaling pathways. More advances in neural networks, including graph and ensemble methods, may hold promise for the discovery of new genes involved in cell signaling pathways.

## 4 AI in Predicting Drug Resistance and Therapeutic Response

### 4.1 ML-Based Prediction of Drug Response and Resistance

One benefit of applying ML methods is the ability to predict a patient’s response to pharmacological treatment. Using pre-existing data on a cancer’s multiomic molecular network and patient outcomes, ML algorithms have been developed to assess a cancer’s susceptibility to a drug and the likelihood of developing drug resistance. Genes encoding cell signaling cascades are a biologically meaningful metric for predicting drug response and prognosis, as they are implicated in a wide variety of cancers (Table 2).

**Table 2:** AI-driven pathway-drug association discoveries in patient dataset analysis.

Drug/ Regimen	Cancer/ Dataset	Targeted Signaling Pathway (s)	AI/ML Model Applied	Predictive Genes/Pathway Associations	Outcomes/ Therapeutic Insights	References
Afatinib, Gefitinib, Nilotinib, Staurosporine, Crizotinib, Lapatinib, Osimertinib, Tamoxifen, Dabrafenib, Leflunomide, Palbociclib, Trametinib, Daporinad, Linistinib, Ribociclib, Ulixertinib, Dasatinib, MK-1775, Ruxolitinib, Vinblastine, Erlotinib, Navitoclax, Sorafenib, Vorinostat	Cancer Cell Line Encyclopedia (CCLE) & Genomics of Drug Sensitivity (GDSC) multi-omics datasets	46 KEGG pathways, including ErbB, Ras, Calcium, FoxO, mTOR, Wnt, Hedgehog, NOD-like receptor, T-cell receptor	consDeepSigna- ling–deep neural network constrained by pathway structure	Integrated gene expression data was combined with data from 791 cell lines; ErbB, Ras, Calcium, FoxO, mTOR, Wnt, and Hedgehog were found to be key predictors of drug response	The pathway-informed Deep Learning model outperformed baseline Deep Neural Networks for anticancer-drug response modeling	[119]
Palbociclib (CDK4/6 Inhibitor)	38 Breast Cancer Cell Lines Including: MCF7, T47D, ZR75-1, BT-474, MDA-MB-231, MDA-MB-361, & SKBR3	Cell-cycle regulation and growth-factor signaling assemblies/Wnt-β-Catenin	NeST-VNN (Visible Neural Network guided by NeST protein assembly map)	Eight core assemblies consisting of 90 gene-linked cell-cycle, growth-factor, and chromatin complexes (KAT6a, TBL1XR1, RUNX1, RB1, and CCND1) to Palbociclib response & resistance	The Assembly-guided model predicted drug response as drivers of CDK4/6i sensitivity and resistance	[120]
Fedratinib & Refamitinib	1000 cell lines across: BRCA, NSCLC, SCLC, CRC, Melanoma, Glioblastoma, Leukemia & Lymphoma Lineages	JAK-STAT, PI3K-AKT, MAPK/ERK	Modular Graph Neural Network (GNN)	Fedratinib sensitivity was linked to JAK-STAT activation, and Refamitinib to MAPK/PI3K co-activation	The GNN successfully captured consistent pathway-drug relationships across multiple kinase inhibitors	[90]
Temsirolimus ± Trametinib	11 Human Multiple Myeloma Cell Lines: NCI-H929, MM.1S, U266, LP-1, KMS-12-BM, KMS-11, RPMI-8226, KMS-18, KMS-28BM, KMS-34, KMS-20	PI3K-AKT-mTOR and MAPK/ERK	Experimental drug-combination study (No AI-Model)	High p-S6 and low p-AKT predicted Temsirolimus response, p-ERK1/2 was further suppressed.	The study found dual mTOR + MEK inhibition produced synergistic proliferative effects independent of cytogenetic subtype	[56]
Palbociclib, Ribociclib, Abemaciclib (CDK4/6 Inhibitors)	HR^+^ HER2^−^ metastatic breast cancer	ER, PI3K, RTK, p53, Cell-cycle	Logistic Regression + Gradient-Boosted Machine (GBM)	ESR1, RB1, NF1, TP53, SMAD4 SNVs; Pathway alterations with highest relative importance (RI) were: ER, p53, PI3K, and RTK SNVs	ML identified ER, RTK, and cell-cycle pathway alterations as key determinants of CDK4/6i resistance and shorter PFS under therapy	[121]

Note: Abb: Artificial Intelligence (AI); Erythroblastic Oncogene B (ErbB); Rat Sarcoma (Ras); Forkhead Box O (FoxO); Mechanistic Target of Rapamycin (mTOR); Cyclin-Dependent Kinase (CDK); Janus Kinase (JAK); Signal Transducers and Activators of Transcription (STAT); Phosphoinositide 3-Kinase (PI3K); Protein Kinase B (AKT); Mitogen Activated Protein Kinase (MAPK); Extracellular Signal-Regulated Kinase (ERK); Machine Learning (ML); Receptor Tyrosine Kinase (RTK); Estrogen Receptor 1 (ESR1); Retinoblastoma 1 (RB1); Neurofibromatosis Type 1 (NF1); Tumor Protein p53 (TP53); Mothers Against Decapentaplegic Homolog 4 (SMAD4); Single Nucleotide Variant (SNV).

One analysis screened cancer samples for mutations in ten common cell-signaling pathways and found that 89% of samples contained at least one aberration [4]. A recent study proposed a new deep learning model, consDeepSignaling, that analyzed expression and copy number data for genes coding 46 cell signaling pathways across 791 cancer cell lines [119]. The proposed deep learning model aimed to identify which pathway genes have the most relevance in predicting therapeutic response, and found that among the 47 signaling pathway genes, ErbB, Ras, Calcium, FoxO, mTOR, Wnt, Hedgehog, NOD-like receptor, and T-cell receptor had the highest significance scores when it came to predictive power [119]. ConsDeepSignaling was validated using a five-fold cross-validation process across all the drugs and cancer cell lines, where high Pearson correlations on test sets, comparative benchmarking, and label-permutation tests showed high robustness [119]. The reproducible prioritization of well-established oncogenic pathways that converge to mediate drug response and resistance successfully linked predictive accuracy to interpretable signaling biology.

In addition to using ML to identify genes with high predictive power, algorithms have been further developed to apply these genes to predict resistance to key cancer drugs, such as kinase inhibitors. For example, cyclin-dependent kinase 4 and 6 inhibitors (CDK4/6i) have been a key part of breast cancer treatment due to their role in preventing cell cycle progression by phosphorylating the retinoblastoma protein [122]. Despite being a first-line drug for hormone receptor-positive, HER2-negative breast cancer (HR+, HER2−), CDK4/6i becomes less effective over time as most patients develop resistance [122]. Recently, a deep learning model was proposed to analyze breast cancer tumors using more than 90 gene signatures associated with cell-signaling pathways to predict resistance to palbociclib, a CDK4/6 inhibitor [120]. The deep learning model found that cells with copy number amplifications in the *MYC*, *TERT*, *KAT6A*, *TBL1XR1*, and *RUNX1* genes were twice as likely to be resistant to palbociclib than cells without such amplifications [120]. These findings are significant for highlighting the role of certain cell-signaling pathways in chemotherapy drug resistance; for example, the *TERT* and *RUNX1* genes are known to activate the Wnt/β-catenin pathway, which is implicated in tumor progression and metastasis [123–125]. The proposed deep learning model provides evidence that links copy number amplifications in Wnt/β-catenin pathway genes to increased drug resistance, thereby offering a new opportunity to fine-tune drug therapies and combat drug resistance.

### 4.2 AI Modeling of Synergistic Drug Therapies

Deep learning models have also been developed to predict resistance across different classes of chemotherapy drugs. A recent study developed CANDELA, a novel graph neural network for estimating cancer drug sensitivity [90]. CANDELA was evaluated in its application to precision oncology, in which a patient’s primary tumor transcriptomic profile is matched to a known drug panel to predict drug sensitivity [90]. The graph neural network was also applied to identify features among 2089 genes that contributed to explaining resistance or drug sensitivity [90]. The algorithm found that genes encoding proteins involved in signaling pathways, primarily JAK/STAT, were highly important for a cell line’s sensitivity to Fedratinib, a highly specific kinase inhibitor [90]. Furthermore, those same cell lines showed increased expression of *FLT3*, *TEK*, and *ERBB2*, all of which are genes significant in PI3K/AKT signaling, suggesting crosstalk between the two pathways [90]. In other cell lines, CANDELA found that genes in the MAPK/ERK signaling pathway were highly important for resistance and sensitivity to Refamitinib, specifically MAP4K1, hepatocyte growth factor (HGF), colony-stimulating factor 1 receptor (CSF1R), and fibroblast growth factor receptor (FGFR2) [90]. Interestingly, cell lines sensitive to Refamitinib also showed significant crosstalk with the PI3K/AKT signaling pathway, with over 30 genes identified [90]. CANDELA was subsequently cross-validated across two clinically relevant scenarios and tested for statistical significance using the Holm-Šídák correction, demonstrating that performance improvements relied on biology-guided elements such as graph attention, modular score decomposition, and pathway-aware pre-training [90]. Discovering evidence of signal crosstalk in a tumor opens the door to developing combination therapies and potentially improving patient outcomes. AI models for predicting drug synergy have already been proposed; for example, a graph neural network, SynerGNet, has been developed to predict the synergistic effect of two anticancer drug regimens and was shown to be more effective than traditional ML algorithms [88]. The graph neural network model lends itself to integrating complex molecular and cellular features within biological systems, enabling it to outperform conventional ML algorithms. Applying these deep learning technologies to cancers that exhibit signaling pathway crosstalk could yield more effective drug therapies.

### 4.3 Integration of Signaling Signatures with Clinical Outcome Data

AI models are valuable in linking key signaling pathways to prognosis and patient outcome. Phosphoproteomics, a promising field within multiomic analysis of tumors, has been instrumental in identifying cell signaling pathways that are actively phosphorylated in tumor cells [126]. Protein phosphorylation is a key post-translational modification that regulates intracellular signaling pathways, ultimately affecting tumor growth, proliferation, and resistance to apoptosis [127]. By capturing dynamic, functional signaling states rather than static genomic alterations alone, these AI-driven phosphoproteomic approaches may provide more accurate and clinically relevant predictions of therapeutic response and resistance. Specifically, two ML algorithms, CoPheeMap and CoPheeKSA, were recently developed to identify activation of key serine/threonine kinases and phosphosites across 1195 tumor samples from 11 cancer types [128]. The ML models revealed hyperactivity of previously underexplored kinases, including CDK12, which plays a major role in regulating *MYC* expression, Wnt/β-catenin signaling, RNA splicing, and PI3K/AKT and ErbB pathways [128,129]. This was validated using independent test datasets, and Monte Carlo cross-validation was performed using extensive pan-cancer Clinical Proteomic Tumor Analysis Consortium (CPTAC) phosphoproteomic datasets [128]. Additional benchmarking using motif- and network-based baselines, along with orthogonal validation, was carried out using experimentally determined kinase-substrate specificity [128]. The biological validity of the results is indicated by the identification of canonical cancer signaling pathways (e.g., CDK, MAPK, PI3K-AKT, Wnt), the clustering of co-regulated phosphosites in known functional pathways, and consistency with tumor-specific kinase activation. Identifying new kinases with aberrant activity is clinically significant, as it offers the chance to explore potential therapeutic targets.

Aberrant activity of kinases within signaling pathways, including hyperactivity, dysfunction, or overexpression, contributes significantly to tumorigenesis and can serve as a powerful marker for identifying signatures linked to patient outcomes and treatment responses [130]. One study used phosphoproteomics to assess the susceptibility of *FLT3*-mutated AML to midostaurin chemotherapy [131]. AML has a poor prognosis, and midostaurin plus intensive chemotherapy (MIC) treatment has been approved only for patients with a mutation in the *FLT3* gene, which encodes a receptor tyrosine kinase involved in STAT5, MAPK/ERK, and PI3K/AKT signaling pathways [132–135]. Although MIC is approved for *FLT3* mutation-positive AML only, some patients still exhibit drug resistance. The study sought to use phosphoproteomics to identify signaling signatures that confer susceptibility to treatment and to explore these signatures as potential predictors of MIC efficacy in patients who are *FLT3* mutation negative [131,136,137]. The ML MIC response-prediction model, named MPhos, identified three distinct subtypes of long-term AML survivors with MIC sensitivity; Group 1 exhibited upregulation of PI3K/AKT, while Group 2 showed increased CK2 and DNA-PK activity, as well as phosphorylation of NHEJ and cohesin proteins. Group 3 exhibited increased phosphorylation of splicing proteins [131]. Associating MIC treatment sensitivity with expression of these three signaling signatures, rather than solely with positive *FLT3* mutation status, may benefit other AML patients who would otherwise not be eligible for MIC treatment. Furthermore, the application of this technology to other drug regimens across various cancer types offers the potential to improve overall patient outcomes.

Another such study used phosphoproteomics to analyze pre-treatment HER2+ early-stage invasive breast cancer to predict response to treatment with trastuzumab, pertuzumab, and chemotherapy [138]. Although these treatments have improved patient outcomes, 20%–40% of patients experience intrinsic or acquired resistance [139]. Phosphoproteomic analysis was used to fill gaps in understanding and to propose cell signatures that confer drug resistance. The study found that no single mechanism predicted drug resistance; rather, a panel of molecular signatures involving the HER2, HER4, ESR, IGF1R, and Kalirin proteins [138]. The data showed that HER4 overexpression could compensate for HER2 inhibition, contributing to treatment resistance [138]. Moreover, elevated phospho-IGF1R expression was implicated in drug resistance, a growth factor typically overexpressed in many cancers, and functions to stimulate PI3K/AKT-mediated differentiation and ERK [138,140,141]. Phosphoproteomic analysis using ML models has the potential to predict key signaling pathways associated with drug resistance, therefore uncovering new potential drug targets that could improve clinical outcomes.

## 5 Current Limitations and Future Directions

### 5.1 Current Limitations

A major challenge in developing accurate ML prediction models remains data heterogeneity and variability, which skews the accuracy of algorithmic outcomes. Termed “batch effects,” the heterogeneity introduced by the technical preparation of data samples must be controlled for [142]. Batch effects can significantly impact the predictive performance of ML models; for example, a study investigating five batches of histopathological images of renal tumors found that batch effects reduced the AI model’s predictive performance [143]. In this case, the batch effect stemmed from the preparation and collection of the specific tumor samples used. To mitigate the inherent variation across tumor batches, a corrective algorithm was developed. By applying the ComBatN algorithm, a feature-level normalization method, the study eliminated batch effects and significantly improved cross-batch validation accuracy in renal tumor prediction models, with a relative increase of 83% and 90% for cross-batch and combined-batch prediction models, respectively, compared with no normalization [143]. Similar batch effect correction algorithms (BECAs) have been developed to mitigate these effects, and a recent study conducted as part of the Quartet Project comprehensively assessed seven BECAs for their ability to reduce batch effects [144]. The study determined that, among the proposed BECAs, the ratio-based approach was most successful across transcriptomics, proteomics, and metabolomics datasets, making it a highly effective and broadly applicable algorithm for reducing batch effects [144]. Although ML methods have been highly informative in multiomics research, more effort is needed to ensure data normalization when using BECAs.

Another challenge in ML multiomics research is the generalizability of models and their applicability to cancer datasets outside those used for training. These challenges, once again, may be attributed to batch effects and the highly heterogeneous nature of multiomics data [145]. A landmark study investigating the distributions of ML performance across cancer types sought to identify the most generalizable model. The study evaluated thousands of models, 4200 for lung adenocarcinoma and 1680 for glioblastoma, to explore how different design factors influence both performance and generalizability, and found that the most influential factors for cross-data success were the selection of features based on differentially expressed genes, or genes that stand out in their expression in cancer vs. healthy tissue samples [146]. The findings demonstrated that biologically informed feature selection, specifically with differentially expressed genes, substantially improved model generalizability across different datasets. This suggests that features reflecting biological processes are less susceptible to batch-specific noise and may be more accurate when applied to cross-cohort studies using ML models. In addition to differentially expressed genes, aberrant expression of cell signaling pathways can be another strong feature for training ML models, thereby improving their predictive power when applied to datasets across different cancer types.

Aside from the mechanistic limitations of AI/ML use, their performance is also limited by factors such as data privacy, reliability, scalability, data representativeness, algorithmic fairness, and the overall inherent heterogeneity of study design [147]. In clinical use with AI/ML frameworks, systemic bias can be embedded in electronic health record-derived data, including missingness, misclassification, and unequal representation of vulnerable populations—a large concern if not explicitly addressed [148].

Data privacy is an essential consideration in oncology, a highly data-intensive domain that relies heavily on sensitive, multi-modal data [147]. Federated learning has so far been successful in enabling multi-institutional model training without exposing raw sensitive data beyond institutional boundaries, though it still faces challenges such as the statistical diversity of data across clients [147,149,150]. As AI/ML frameworks evolve, source-free domain adaptation (SFDA) and blockchain technology have offered solutions to preserving patient privacy and maintaining AI model integrity [147]. SFDA aims to adapt a well-trained model from a source domain to target domains that don’t have access to source data or require target-domain labels [151]. Blockchain technology, meanwhile, employs a distributed ledger system that ensures patient records remain immutable and verifiable, providing a secure, decentralized method for managing patient health data [152].

Reliability and scalability are central to AI/ML application translational research, as code sharing for AI models is crucial for reproducibility and clinical application [147,153]. Models must be independently reproducible, although most studies validate them using external datasets [147]. A further limitation in contemporary research on reproducibility is the disparity in data acquisition across platforms and the accessibility of that data to other institutions, particularly for private datasets [147,154]. Moreover, these AI/ML models are often applied to large datasets such as TCGA. The repeated overreliance on a select few large-scale resources, such as the TCGA, further increases the risk of overfitting and limited reproducibility, as deep learning models may learn site-specific or non-biological features that inflate performance yet fail to generalize across other independent cohorts [155]. This overuse of the TCGA also affects data representativeness, as such models remain fundamentally biased towards certain racial groups [147]. The TCGA, a landmark and largest oncological dataset, has a median European ancestry of 83%, and a 2016 genomic analysis also showed it is insufficient for representing all ethnic minority populations [147,156,157]. Beyond genomics, commercial imaging datasets on which AI models heavily rely also lack diversity in race, geography, and socioeconomic status, thereby systematically overlooking rural or low-income populations [147]. This inadvertently affects algorithmic fairness, as systematic disparities influence model performance metrics, including accuracy, false-negative rates, and calibration across subgroups defined by social determinants of health [158,159]. Overall, interpretability and clinical feasibility remain concerns, as opaque AI systems can obscure biological rationale and complicate accurate real-world use, emphasizing a need to balance predictive performance with reproducibility and bias mitigation [160,161].

### 5.2 Future Directions

**Figure 2:**
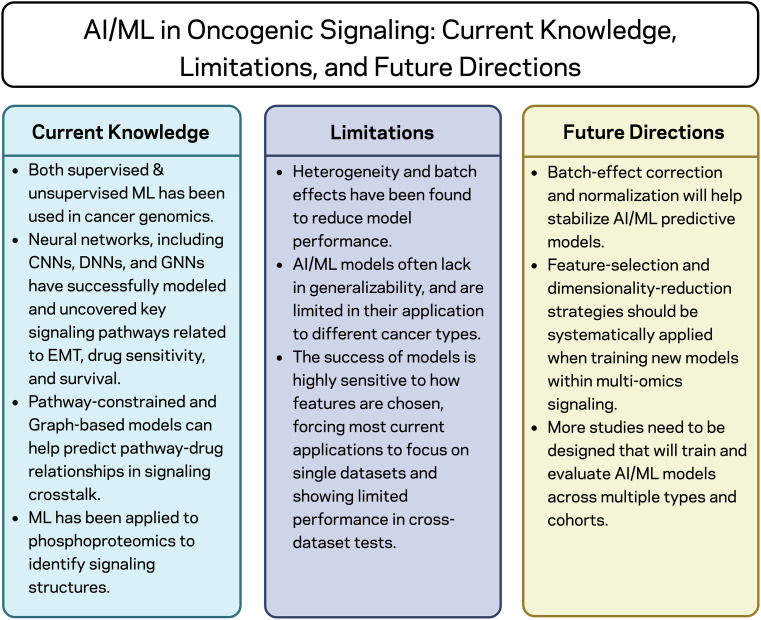
Artificial intelligence (AI) and machine learning (ML) in oncogenic signaling: current knowledge, limitations, and future directions. AI and ML have advanced our understanding of cancer signaling by modeling complex pathways and identifying key molecular interactions. However, persistent challenges, such as data variability, limited generalizability, and sensitivity to feature selection, continue to limit the reliability of the models. Addressing these through enhanced data normalization, feature optimization, and cross-dataset validation will further strengthen performance and expand AI/ML’s clinical application in oncology. Created in BioRender.com. Stojchevski R. (2025) https://BioRender.com/fwucaib [162].

Ultimately, several important knowledge gaps remain despite the rapid advances in AI and ML approaches to modeling oncogenic signaling pathways (Fig. 2). First, current research often relies heavily on correlations derived from high-dimensional data, and few studies validate their results experimentally to ensure that modeled signaling pathways are biologically significant in cancer. Second, current models often fail to capture the dynamic and context-dependent properties of feedback and compensatory activation of signaling pathways because they are typically trained on genomic or transcriptomic data that reflect fixed cellular states rather than signaling pathway function or dynamics. Third, there is currently no standardized framework that combines AI predictions with perturbation-based experimental systems, such as pharmacologic inhibition or knockdown, to repeatedly validate and improve AI predictions. Lastly, very few research efforts focus on translating AI predictions of signaling interactions into practical therapeutic strategies, particularly those that guide the design of combination therapies.

## 6 Conclusion

The interplay among major oncogenic signaling pathways remains a frontier for better understanding tumorigenesis and oncological therapeutics. Rather than functioning in isolation, these pathways are deeply interconnected signaling networks that ultimately drive tumor cell proliferation, survival, metastasis, and drug response. By understanding oncogenesis through this network-based lens and by evolving computational models, a better framework emerges for interpreting tumor adaptability and resistance, compared with traditional linear and reductionist models.

AI/ML frameworks are not merely data-analysis tools; they serve as transformative engines that uncover the hidden logic of tumor adaptability, thereby directly guiding the design of combinatorial therapies and overcoming resistance mechanisms. By modeling nonlinear, context-dependent signaling interactions across multiomics datasets, AI/ML frameworks enable insights that exceed the capacity of traditional reductionist approaches. These applications have provided the bridge between mechanistic biology and clinical translation, offering revolutionary ways to refine therapeutic strategies tailored to cancer signaling. The insights developed by these systems will enable the design of multi-target therapeutic regimens that can disrupt these aberrant interwoven networks.

However, it is important to recognize the current limitations of these models, with batch effect and the generalizability of ML models across cohorts and different cancer types being the most prevalent. ML models’ ability to learn from crosstalk is hindered by heterogeneity in data and sample collection, which contributes to noise and limits cross-cancer generalizability, since learning from a single dataset may be less successful when applied to other cancer types or datasets. Future progress depends on integrating these AI/ML predictions with mechanistic experimental perturbations while enhancing reproducibility, interpretability, scalability, and representativeness to more effectively translate findings into robust clinical therapeutic strategies. Nonetheless, the convergence of AI models and signaling biology marks a unique era in which computational power can be leveraged to transform precision oncology, guided by systems-level understanding rather than isolated pathway targeting.

## Data Availability

Not applicable.

## Abbreviations

AI: Artificial Intelligence
ML: Machine Learning
MAPK: Mitogen-Activated Protein Kinase
RTK: Receptor Tyrosine Kinase
PIP_2_: Phosphatidylinositol 4,5-triphosphate
PIP_3_: Phosphatidylinositol (3,4,5)-triphosphate
FZD: Frizzled Receptor
NICD: Notch Intracellular Domain
TNBC: Triple Negative Breast Cancer
EMT: Epithelial-to-Mesenchymal Transition
PI3K: Phosphoinositide-3-Kinase
AKT: Protein Kinase B
JAK: Janus Kinase
STAT: Signal Transducer and Activator of Transcription
HNSCC: Head and Neck Squamous Cell Carcinoma
NF-κB: Nuclear Factor Kappa-Light-Chain-Enhancer of activated B cells
TNF: Tumor Necrosis Factor
ROC: Receiver Operating Characteristic
AUC: Area Under the Curve
PR: Precision Recall
GNN: Graph Neural Network
CNN: Convolutional Neural Network
DNN: Deep Neural Network
HPV: Human Papillomavirus
VAE: Variational Autoencoder
CCLE: Cancer Cell Line Encyclopedia
GDSC: Genomics of Drug Sensitivity
VNN: Visible Neural Network
GBM: Gradient Boosted Machine
CFL1: Actin-Binding Protein Cofilin
MNIST: Modified National Institute of Standards and Technology
RASA1: Ras GTPase-activating Protein 1
ErbB: Erythroblastic Oncogene B
Ras: Rat Sarcoma
FoxO: Forkhead Box O
mTOR: Mechanistic Target of Rapamycin
ERK: Extracellular Signal-Regulated Kinase
ESR1: Estrogen Receptor 1
RB1: Retinoblastoma 1
NF1: Neurofibromatosis Type 1
TP53: Tumor Protein p53
SMAD4: Mothers Against Decapentaplegic Homolog 4
SNV: Single Nucleotide Variant
CDK4/6i: Cyclin-Dependent Kinase 4 and 6 Inhibitors
HGF: Hepatocyte Growth Factor
CSF1R: Colony Stimulating Factor 1 Receptor
FGFR2: Fibroblast Growth Factor Receptor
AML: Acute Myeloid Leukemia
TCGA: The Cancer Genome Atlas
MIC: Midostaurin Plus Intensive Chemotherapy
BECA: Batch Effect Correlation Algorithm
CPTAC: Clinical Proteomic Tumor Analysis Consortium
SFDA: Source-Free Domain Adaptation
